# BAG3 induces α‐SMA expression in human fibroblasts and its over‐expression correlates with poorer survival in fibrotic cancer patients

**DOI:** 10.1002/jcb.30171

**Published:** 2021-11-06

**Authors:** Margot De Marco, Nicoletta Del Papa, Francesca Reppucci, Vittoria Iorio, Anna Basile, Antonia Falco, Roberta Iaccarino, Sergio Brongo, Francesco De Caro, Mario Capunzo, Maria Caterina Turco, Alessandra Rosati, Liberato Marzullo

**Affiliations:** ^1^ Department of Medicine, Surgery and Dentistry Schola Medica Salernitana University of Salerno Baronissi Salerno Italy; ^2^ R&D Division BIOUNIVERSA s.r.l. Baronissi Salerno Italy; ^3^ Rheumatology Department, Scleroderma Unit G. Pini Hospital Milano Italy

**Keywords:** BAG3, fibrotic tumor, myofibroblasts, smooth muscle actin

## Abstract

Hypoxia and angiogenesis in solid tumors are often strictly linked to the development of fibrotic tissues, a detrimental event that compromises the antitumor immunity. As a consequence, tumor aggressiveness and poor patient prognosis relate to higher incidence of tissue fibrosis and stromal stiffness. The molecular pathways through which normal fibroblasts are converted in cancer‐associated fibroblasts (CAFs) have a central role in the onset of fibrosis in tumor stroma, thus emerging as a strategic target of novel therapeutic approaches for cancer disease. Several studies addressed the role of BAG3 in sustaining growth and survival of cancer cell and also shed light on the different mechanisms in which the intracellular protein is involved. More recently, new pieces of evidence revealed a pivotal role of extracellular BAG3 in pro‐tumor cell signaling in the tumor microenvironment, as well as its involvement in the development of fibrosis in tumor tissues. Here we report further data showing the presence of the BAG3 receptor (Interferon‐induced transmembrane protein [IFITM]‐2) on the plasma membrane of normal dermal fibroblasts and the activity of BAG3 as a factor able to induce the expression of α‐smooth muscle actin and the phosphorylation of AKT and focal adhesion kinase, that sustain CAF functions in tumor microenvironment. Furthermore, in agreement with these findings, *bag3* gene expression has been analyzed by high throughput RNA sequencing databases from patients‐derived xenografts. A strong correlation between *bag3* gene expression and patients' survival was found in several types of fibrotic tumors. The results obtained provide encouraging data that identify BAG3 as a promising therapeutic target to counteract fibrosis in tumors.

## 1 INTRODUCTION

Many types of tumors produce abnormal fibrotic shells made of fibroblasts, immune cells, and dense extracellular matrix (ECM). The formation of such a fibrotic tumor microenvironment (TME) causes a limited and unsatisfactory outcome of pharmacological therapies.^1^ Moreover, especially in pancreatic adenocarcinomas (PDAC), this environmental constraint prompts the neoplastic cells to survive in low oxygen/low nutrient conditions, thus forcing their metabolism to develop an aggressive and drug‐resistant phenotype. Among the cells populating the TME of PDAC, cancer‐associated fibroblasts (CAFs) can particularly support pancreatic cancer cell proliferation under harsh metabolic stringencies.^2^ Conventional therapeutic protocols, including treatments with chemotherapeutics, imply several side effects and adverse and off‐target events, that reduce their use and efficacy and often also impose the selection of subsets of responsive patients. Therefore, a great deal of effort is nowadays put into research aimed at developing new and improved therapeutic approaches having higher specificity and lower risk of side effects, and able to consider the current awareness about the great biological heterogeneity of cancer types and subtypes, the challenges faced in harnessing the metastatic processes, and the related variable responses of nontargeted selection of patients to conventional therapies.^3^

It is believed that strategies including anti‐fibrotic treatments could be of great help in the attempt to normalize the TME, and CAFs—the main producers of ECM and paracrine signals—represent a lead target for innovative therapeutic approaches.^4^

It has been demonstrated that BAG3 protein can be secreted by some cell types, in particular, pancreatic adenocarcinoma cells and cardiomyocytes, under oxidative stress.^5^, ^6^ Moreover, it has been shown that BAG3 secreted by pancreatic tumor cells induces the activation of Tumor‐Associated Macrophages (TAMs) through the binding to its receptor Interferon‐induced transmembrane protein (IFITM)‐2, which in turn activates p38 and PI3K and stimulates the production of molecules, such as interleukin (IL)‐6, that support the neoplastic growth. A monoclonal antibody able to bind extracellular BAG3 can block its activity and to impair the tumor growth and the metastatic process in three different PDAC mouse models, thus confirming the relevance of the protein in the neoplastic cells—TAMs cross talk.^7^, ^8^ Several pieces of evidence suggest a close signaling connection between CAFs and TAMs in the tumor stroma: for example, alpha‐smooth muscle actin (α‐SMA)‐expressing fibroblasts have been proved to effectively recruit monocytes.^9^ It is worth noting that in two different PDAC animal models (allografts of a murine pancreatic cancer cell line in syngeneic mice and xenografts of patient‐derived PDAC in immunodeficient mice) the treatment with an anti‐BAG3 monoclonal antibody produced a strong down‐modulation of the expression of α‐SMA—an activation marker of CAFs—and a marked reduction of collagen deposition.^10^ Then the available evidence let infer a close correlation of BAG3 expression with the cancer fibrotic phenotypes, responsible for the production of mechanical forces and the establishment of a biochemical milieu that finally affect the intratumoral immunity and influence the metastatic behavior of tumor cells (see Refs. 11, 12, 13). The results of our investigation here reported highlight the potential role of BAG3 in the fibrogenesis of tumor stroma and show how the analysis of *bag3* expression could be exploited as a marker of disease progression in patients affected by fibrotic tumors.

## 2 MATERIALS AND METHODS

### 2.1 Cloning and expression of recombinant BAG3

Human recombinant BAG3 protein was produced as previously described by Gok Yavuz et al.^14^ Briefly, human *bag3* CDS (Accession Number NM_004281.3) was chemically synthesized (GenScript) after gene analysis and optimization for expression in *Escherichia coli* with OptimumGeneTM software (GenScript). The synthetic DNAs fragments, adapted at 5ʹ and 3ʹ ends, were cloned into the pAViTag‐N N‐His SUMO Kan Vector (Lucigen, #49044‐1) and used to transform *E. coli* Biotin XCell Fʹ cells (Lucigen, #0704‐1). The recombinant protein carried a fused N‐terminal biotinylated tag that allowed its capture on streptavidin agarose resin (Thermo Scientific, #20359). The subsequent on‐column cleavage with SUMO Express Protease (Lucigen, #30801‐2) released the full‐length protein that was further purified on NTA‐Ni resin (Sigma‐Aldrich; #P6611) to remove the His‐tagged protease. Pierce High‐Capacity Endotoxin Removal Spin Column (Pierce, #88274) was used to obtain endotoxin‐free preparation. Endotoxin concentration was measured by QCL‐1000™ Assay (LONZA, #50‐647U) following the manufacturer's instructions. The purification protocol allowed to obtain recombinant protein preparations >95% pure, <3 EU/mg endotoxins. rBAG3 FITC conjugation was performed using FluoroTag FITC conjugation kit (FITC1‐1KT) following the manufacturer's instructions.

### 2.2 Cell cultures

For this study, normal human dermal fibroblasts (NHDF; Lonza Bioscience) or human dermal fibroblasts (HF; CELL Application, Inc) were used and cultured in a specific medium provided by manufacturers. On receipt, cells were grown at 37°C in a 5% CO_2_ atmosphere, expanded, cryopreserved as low‐passage stocks, and tested routinely for mycoplasma immediately before use in an experiment.

### 2.3 FACS binding assay

NHDF cells (up to 1 × 10^6^ ml^−1^) were incubated with 80 µl of 1X PBS containing 10% heat‐inactivated fetal bovine serum (FBS), 0.1% NaN_3_ (binding buffer), and 20 µl of FcR Blocking Reagent (Miltenyi Biotec) for 15 min on ice, following manufacturer's instructions (cat. No. 130‐059‐901), then suspended in the binding buffer and incubated for 15 min on ice. Thereafter, the staining was carried out in binding buffer by incubating the cells for 30 min on ice with an in house produced FITC‐conjugated anti‐FITM‐2 monoclonal antibody, or with an unrelated mouse IgG1‐FITC (Santa Cruz Biotechnology; sc‐2866) as a negative control. For competition assays, cells were pretreated with rBAG3 1× (10 μg/ml) and 10× (100 μg/ml), or with bovine serum albumin (BSA) used as an unrelated control, for 30 min on ice and then 20 μg/ml of the FITC‐conjugated anti‐IFITM‐2 monoclonal antibody was added to the mixture and incubated for additional 30 min on ice. After incubation, cells were washed with PBS/2% FBS/0.1% NaN_3_, centrifuged for 10 min at 300 g, resuspended in 300 µl of binding buffer, and analyzed by flow cytometry, using FACSVerse Flow Cytometer (BD Biosciences). The antibody FITC‐conjugation was performed using a FluoroTag FITC conjugation kit (FITC1‐1KT) following the manufacturer's instructions.

### 2.4 Immunofluorescence

NHDF cells were cultured on coverslips in six‐well plates to 60%–70% confluence; after 16 h treatment, coverslips were washed in 1× PBS and fixed in 3.7% formaldehyde in 1× PBS for 30 min at room temperature, and then incubated for 5 min with 1× PBS‐0.1 M glycine. After washing, coverslips were permeabilized with 0.1% Triton X‐100 for 5 min, washed again, and incubated with blocking solution (10% normal goat serum in 1× PBS) for 1 h at room temperature. Following incubation at 4°C overnight with an antibody anti‐α−SMA (A2547, Sigma‐Aldrich; 1:350), coverslips were washed three times with 1X PBS. After incubation with secondary antibodies at room temperature for 45 min, coverslips were again washed for three times in PBS and then in distilled water. Nonpermeabilized NHDF cells were also incubated with an anti‐IFITM‐2 antibody (LS‐C215215‐PE, LSBio; 1:100) and a FITC‐conjugated rBAG3 following the same protocol. The coverslips were then mounted on a slide with interspaces containing mowiol. Samples were analyzed using a confocal laser scanning microscope (Leica SP5, Leica Microsystems). Images were acquired in sequential scan mode by using the same acquisitions parameters (laser intensities, gain photomultipliers, pinhole aperture, objective 63×, zoom 2) when comparing experimental and control material. For preparing the figures, the brightness and contrast of images were adjusted to leave a light cellular fluorescence background, for a better appreciation of the lowest fluorescence intensity and to allow a better comparison among the different experimental groups. Figures were assembled using Adobe Photoshop 7 and Adobe Illustrator 10.

### 2.5 Western blot

Cells were harvested in a buffer containing 20 mM HEPES (pH 7.5), 150 mM NaCl, 0.1% Triton (TNN buffer), protease inhibitors cocktail (Sigma‐Aldrich), and lysed by freeze/thawing (three cycles). Lysates were then centrifuged for 20 min at 15,000*g* and the cleared supernatants were stored at −80°C. Protein concentration was determined by Bradford assay (Bio‐Rad) and 10 µg of total protein were separated on 10% SDS‐PAGE gels and electrophoretically transferred onto nitrocellulose membrane. Nitrocellulose blots were blocked with 10% nonfat dry milk in TBST buffer (20 mM Tris‐HCl at pH 7.4, 500 mM NaCl and 0.01% Tween), and incubated with primary antibodies in TBST containing 5% nonfat dry milk overnight at 4°C. Anti‐α−SMA (A2547; Sigma‐Aldrich), anti‐Hsc70 (Ab90554; Abcam) anti‐phospho‐AKT (#9271, Cell Signaling) and anti‐phospho‐focal adhesion kinase (FAK) (#3283, Cell Signaling) antibodies were used at a 1:1000 dilution. Immunoreactivity was detected by sequential incubation with peroxidase‐conjugated secondary antibodies (used at 1:5000 dilution) and ECL detection reagents (Amersham Life Sciences Inc.). Signal detection was performed using an ImageQuant™ LAS 4000 (GE Healthcare).

### 2.6 Database analyses

We analyzed three different databases containing high throughput RNA sequencing (RNAseq) information from patients‐derived xenografts (PDXs)^15^, ^16^, ^17^, ^18^ Hereafter, the URLs linking to the different data set used for the analyses: database 1 (https://database.championsoncology.com/), database 2 (https://www.crownbio.com/oncology/in-vivo-services/patient-derived-xenograft-pdx-tumor-models), and database 3 (https://www.pdxfinder.org/source/crl/). We collected *bag3* mRNA expression data for all the different tumor types available. The *bag3* mRNA reads obtained from patients‐derived tumors were aligned on the human genome reference (hg19 assembly, UCSC).

Data obtained were expressed as average of *bag3* log2 FPKM (fragments per kilobase per million mapped reads) or RPKM + 1 (reads per kilobase of transcript per million mapped reads +1) for each tumor type and plotted using excel xy distribution graph. The correlation coefficient for *bag3* mRNA expression within different databases for all tumor types was calculated by using GraphPad Prism software and Spearman correlation.

The same databases were used to compare the percentage of samples per tumor type obtained from mice model engrafted with different patient‐derived cancers, presenting amplification (i.e., >2 gene copies) or deletion (i.e., <2 gene copies). Kaplan–Meier survival curves for different cancers were elaborated considering two groups of subjects characterized by low *bag3* expression and high *bag3* expression regarding the median *bag3* expression value. Data were obtained considering the overall patients' survival and using gepia2 database (http://gepia2.cancer-pku.cn/#dataset) containing datasets for HNSCC, pancreatic adenocarcinoma, mesothelioma, and liver hepatocellular carcinoma.

## 3 RESULTS

### 3.1 Extracellular BAG3 induces α‐SMA expression in human normal fibroblasts

**Figure 1:**
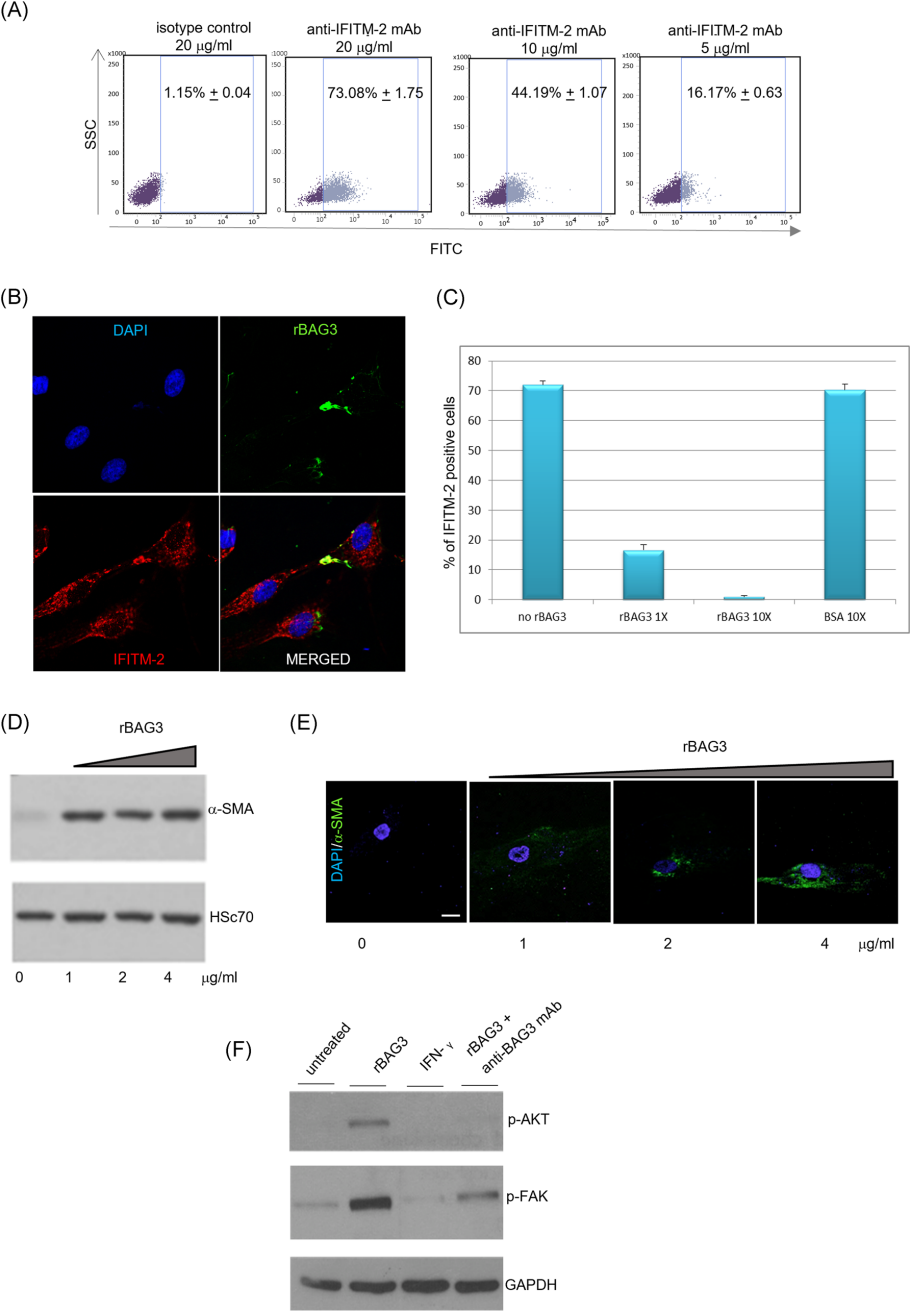
(A) Human dermal fibroblast (HF) cells were seeded at a density of 250,000 cells per well in a six‐well plate. The day after, the cells were analyzed by FACS to measure the expression of IFITM‐2 on their surface using a FITC‐ conjugated anti‐IFITM‐2 antibody produced in our laboratory. Percentage of positive cells (±SD) are displayed in the resulting dot plots. Unrelated FITC‐conjugated IgG1 was used as a negative control. (B) Normal human dermal fibroblast (NHDF) were seeded at a density of 70,000 cells/well in a 24‐well plate on coverslips and incubated at 4°C with FITC‐rBAG3 and with PE‐conjugated anti‐IFITM‐2 antibody (LSBio). A DAPI solution was used to visualize nuclei. Images were acquired using a confocal laser scanning microscope. (C) HF cells were seeded at a density of 250,000 cells per well in a six‐well plate. The day after, the cells were analyzed by flow cytometry using a FITC‐ conjugated anti‐IFITM‐2 antibody (20 μg/ml) and rBAG3 (1× or 10×) bovine serum albumin (BSA) was used as an unrelated control (D) NHDF cells were seeded at a density of 70,000 cells per well in a 24‐well plate and incubated with recombinant (r) BAG3 at the indicated concentrations, for 16 h. After cell lysis and centrifugation, the whole extracts were analyzed by western blot analysis using anti‐alpha‐SMA and anti‐HSC70 antibodies. (E) NHDF were seeded at a density of 70,000 cells/well in a 24‐well plate on coverslips and incubated with recombinant (r) BAG3 at the indicated concentration, for 16 h. α‐SMA expression was analyzed by immunofluorescence using an anti‐α‐SMA antibody. A DAPI solution was used to visualize the nuclei. Images were acquired using a confocal laser scanning microscope. (F) Cells were treated for 16 h with rBAG3 (4 μg/ml) alone or in presence of an anti‐BAG3 antibody. Cells harvested after 16 h of IFN‐γ (10 ng/ml) treatment were used as unrelated controls. Whole‐cell extracts were analyzed by western blot analysis using anti‐phospho‐AKT and anti‐phospho‐FAK polyclonal antibodies; an anti‐GAPDH antibody was used as a loading control. DAPI, 4′,6‐diamidino‐2‐phenylindole; IFN, interferon

The presence of the BAG3 receptor on the CAF cell surface was previously hypothesized based on the results obtained in a study on pancreatic cancer murine preclinical models.^10^ Indeed, that evidence suggested that extracellular BAG3 is involved in the activation pathway which leads to differentiation of fibroblasts into myofibroblasts, which in turn contribute to tumor fibrosis development. To demonstrate the possible direct interaction of extracellular BAG3 (eBAG3) with fibroblasts, the expression of the BAG3 receptor IFITM‐2 on HF cell surface was verified by fluorescence‐activated single cell sorting (FACS) analysis. An in house produced anti‐IFITM‐2 monoclonal antibody was labeled with fluorescein isothiocyanate (FITC) and used to analyze nonpermeabilized human fibroblasts. Figure 1A shows that IFITM‐2 protein is expressed on the cell surface of fibroblasts, as demonstrated by FACS analysis of anti‐IFITM‐2 mAb binding to the cells. Furthermore, as previously shown in murine macrophages and in human monocytes,^8^ the binding of eBAG3 onto fibroblasts plasma membrane was also demonstrated by confocal microscopy using a (FITC)‐conjugated recombinant (r) BAG3 (Figure 1B). In addition, it was detected a cell surface colocalized signal when FITC‐rBAG3 was incubated with a commercial anti‐IFITM‐2 Ab labeled with phycoerythrin and strong inhibition of the anti‐IFITM‐2 mAb binding in FACS analysis when rBAG3 was added in the staining mixture. In particular, the BAG3/anti‐IFITM‐2 equimolar mixture resulted in a ∼75% signal decrease, while a 10× molar excess of BAG3 completely displaced the binding of anti‐IFITM‐2 to its epitope, thus demonstrating a BAG3‐IFITM‐2 specific interaction.

The eBAG3‐induced signaling in cells expressing BAG3R involves AKT phosphorylation,^8^ which in turn is able to trigger downstream the expression of α‐SMA.^19^ To verify the possible activity of eBAG3 on human fibroblasts, NHDF cells were incubated with rBAG3 and the effect on the expression of the fibroblasts' activation marker α‐SMA was then evaluated. As shown in Figure 1D,E, the level of α‐SMA increased in human fibroblasts treated with increasing concentrations of rBAG3 for 16 h. Interestingly, Li and colleagues reported that BAG3 signal was positively correlated with αSMA staining as demonstrated by immunohistochemistry on tissues specimens of pancreatic cancer patients. In the same patients, the extension of fibrosis was monitored by Masson's staining. It is worth noting that in the same paper, the authors reported that BAG3 knockdown in cancer cells favors the recruitment of Argonaute 2 (Ago2) to IL‐6 mRNA, which results in the IL‐6 mRNA destabilization and finally in the reduction of fibrosis onset. To our knowledge, to date no evidence of the involvement of eBAG3 in fibroblast activation was previously reported.

To provide essential signals for cancer cell survival and proliferation, fibroblasts also facilitate cancer cell local invasion and metastatic phenomena. An important role is exerted by FAK signaling, which is activated by the binding of collagen I to integrin α2β1. FAK serves as a scaffolding protein and integral component of focal adhesions is anchored through paxillin and regulates its function by phosphorylation. Other than playing a role in motility regulation, FAK sustains cancer cell survival by more than one mechanism, including its interactions with PI3K and TP53.^20^ FAK activation analysis in rBAG3‐ stimulated cells revealed also that the protein induced the phosphorylation of FAK, as proved by the effect of an anti‐BAG3 mAb, that inhibits eBAG3‐ induced FAK modification (Figure 1F).

### 3.2 BAG3 expression in fibrotic tumors from PDZ models

In the last decade, PDX models played a pivotal role in the progress of basic knowledge and in the development of routine protocols used in the translational research industry. The main advantage mostly consists in the fact that PDX models retain the principal histologic and genetic characteristics of their donor tumor^16^, ^21^, ^22^; on the other hand, a major disadvantage is represented by the absence of interaction between cancer cells (or other microenvironment components) and immune cells, thus compromising the evaluation of drug efficacy and drug resistance mechanisms.^23^ To date a great improvement in PDX model was achieved with the successful creation of humanized models of PDX^24^ that are being used for the development of personalized medicine strategies.

As fibroblasts were shown to be a target of eBAG3, along with the previous evidence of a close relationship of eBAG3 with the development of fibrosis in cancer tissues, we queried some available databases containing high throughput RNA sequencing information from PDXs to highlight possible correlations of BAG3 expression with tumor types and their histological characteristics.^15^, ^16^, ^17^, ^18^

**Figure 2:**
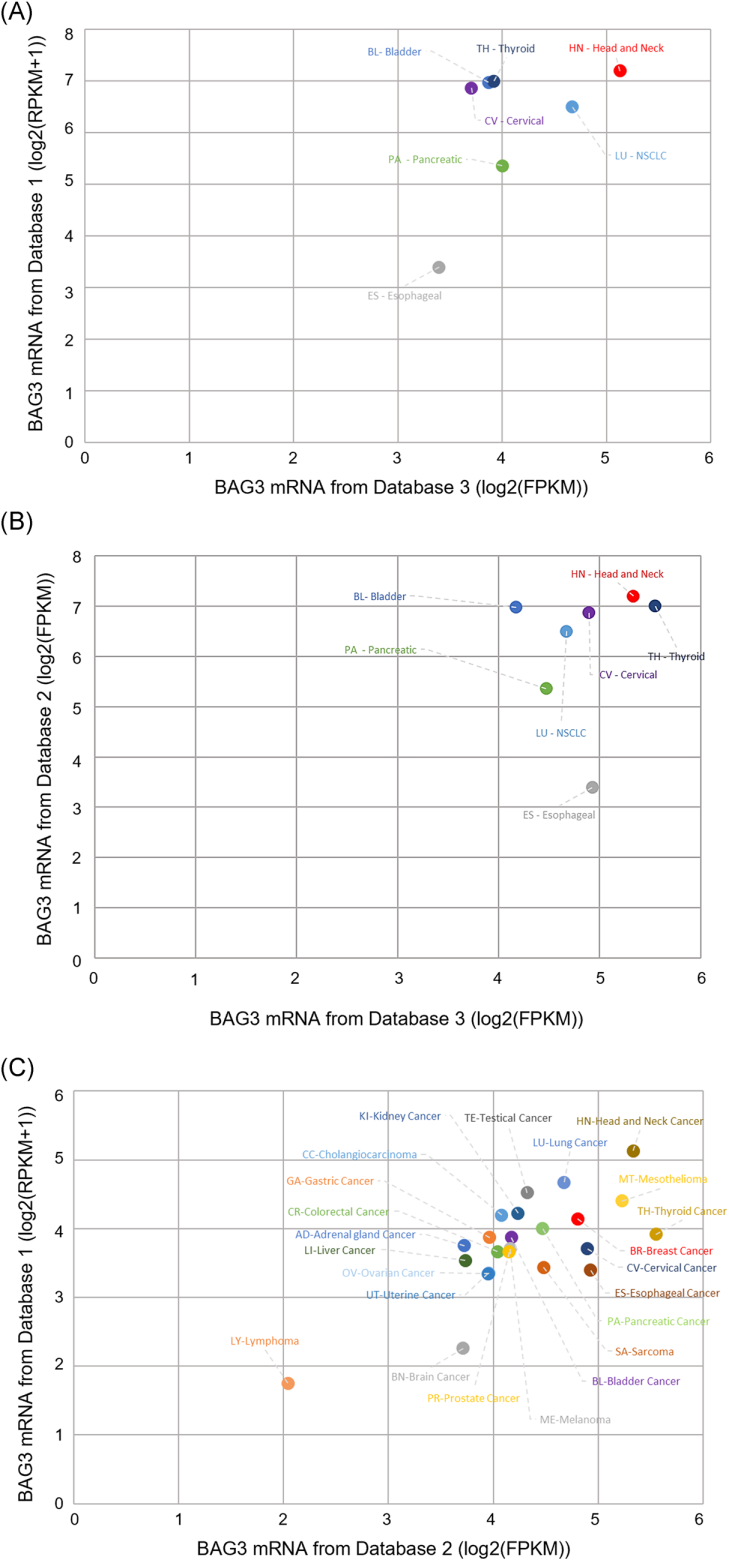
Query results of three databases containing high throughput RNA sequencing information from patients‐derived xenografts. Data correlations with BAG3 levels in different tumor types are reported. (A) Results obtained from databases 1 and 2 are represented as a function of the log2 *bag3* expression averages clustered by tumor types. (B) Results as (A) obtained from databases 1 and 3 data. (C) Results as (A) obtained from databases 2 and 3 data. Results are expressed as an average of *bag3* log2 FPKM (fragments per kilobase per million mapped reads) or RPKM + 1 (reads per kilobase of transcript per million mapped reads +1) for each tumor type and plotted using excel xy distribution graph

Figure 2 shows the comparison of the *bag3* mRNA levels in different PDXs as collected from the three queried databases. The analyzed datasets show a higher expression of BAG3 in head and neck cancer, thyroid cancer, metastatic melanoma, bladder cancer, pancreatic cancer, mesothelioma, NSCLC, esophageal cancer, cervical cancer, lung cancer, and breast cancer, if compared with other nonfibrotic cancer types, such as lymphomas (*p* < .001; *R* = 0.72) (Figure 2C). Head and neck cancer showed particularly high *bag3* mRNA levels, then likely confirming this characteristic as a signature of fibrotic solid tumors.

### 3.3 BAG3 expression and survival of patients affected by different fibrotic tumor types

**Figure 3:**
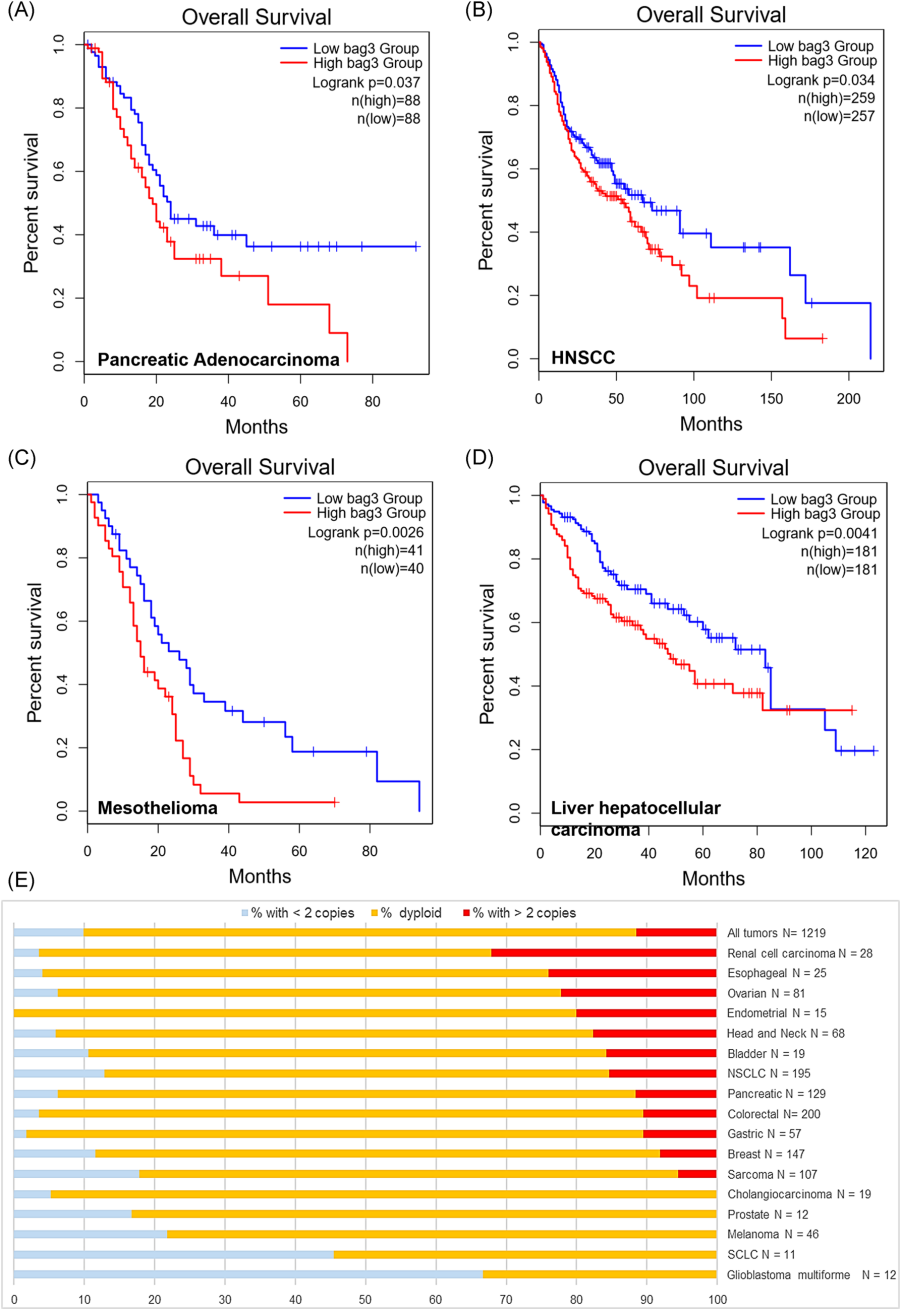
Kaplan–Meier survival curves were generated considering the overall survival of subjects for each type of cancer and clustered in high and low *bag3* expressing groups. Kaplan Meyer curves for (A) pancreatic cancer patients (*N* = 176); (B) head and neck squamous cell carcinoma patients (*N* = 414); (C) mesothelioma patients (*N* = 81); (D) liver cancer patients (*N* = 362). (E) The BAG3 gene copy number was extracted from databases collecting data obtained from mouse xenografts of different human patient‐derived cancer samples. The incidences of tumors presenting a *bag3* gene amplifications (>2 copies) or deletions (<2 copies) were reported as percentage for each tumor type, along with the number of the analyzed samples per tumor type

A growing literature body is emerging on the activities of CAFs in different tumor types, and research efforts are focused onto the development of therapeutic agents able to switch activated fibroblasts into a more resting phenotype.^25^ Forfurther looking at other eligible tumors, other than pancreatic cancer, for anti‐BAG3‐ based therapeutics, a database containing both gene expression data and patients' outcome^26^ was used to find out possible correlations between *bag3* levels in the tumor specimens and patient's overall outcome. As shown in Figure 3, high *bag3* expression is significantly and positively correlated to the fibrotic characteristic of the tumor types analyzed. A clear example of this correlation is represented by the head and neck squamous cell carcinoma (HNSCC) and pancreatic cancer, where an increased CAFs density has been associated with a worse clinical prognosis.^27^, ^28^ A poorer rate of patients' survival in pancreatic cancers expressing high levels of BAG3 protein and *bag3* mRNA was previously reported,^6^ and here confirmed by a distinct analysis data set (*p* = .032) (Figure 3A); similar results were obtained analyzing *bag3* expression in HNSCC, where patients with low *bag3* expression showed longer overall survival if compared to patients with higher BAG3 expression (*p* = .034) (Figure 3B). A further significant and coherent correlation with *bag3* levels was found analyzing the survival data of patients affected by mesothelioma. Studies carried out in preclinical models of mesothelioma demonstrated that CAFs contribute to tumor growth and resistance to therapy mainly by inhibiting cytotoxic T cell influx in the tumor tissue and that the use of chimeric antigen receptor‐transduced T cells targeted to cells expressing fibroblast activation protein (FAP) can reduce the tumor growth in an animal model.^29^, ^30^

Finally, the same database queries were used to analyze the data of liver hepatocellular cancer patients with similar results (*p* = .0041) (Figure 3D). Previous studies in other tumor types, such as hepatocellular cancers, showed that a reduction of the peritumoral fibrotic stroma can effectively contribute to overcome resistance to therapy.^31^ This evidence is also in good agreement with a previous report showing the value of BAG3 levels as a prognostic factor in tumors, in a smaller‐sized study.^32^

### 3.4 *bag3* gene amplification and deletions in different PDXs

An aspect, possibly affecting tumor biology, less considered so far, is the effect of the *bag3* gene copy number variation (CNV) in different tumors. Several pieces of evidence have been reported a pro‐tumor effect of BAG3 in several cancers, while its silencing has a detrimental impact both in tumoral cell growth and metastatic potential.^33^, ^34^ The analysis of genomic PDX datasets allowed us to show the presence of *bag3* CNV (deletions and amplifications) in several tumor types. The results show that fibrotic solid tumors, such as renal cell carcinoma, esophageal cancer, ovarian cancer, endometrial cancer, head and neck cancer, bladder cancer and pancreatic cancer, have a high amplification rate of *bag3* gene (Figure 3E). On the other hand, glioma multiforme and small cell lung cancer (SCLC) show a high incidence of *bag3* gene deletions. The correlation between *bag3* gene amplification, its expression and the amount of BAG3 protein secreted in the microenvironment milieu open new research perspectives, which deserve a more in‐depth investigation, to hopefully provide new hints for the development of therapeutic tools based on BAG3 biochemistry and its role in tumor biology.

## 4 DISCUSSION

Our current knowledge of the heterogeneity of cancer types provides us with a sound awareness about not extendible efficacy of available and developing therapeutics not only to all types of cancers but also indiscriminately to all subjects. In fact, certain cancer types are particularly difficult to treat. Some subjects are not responsive to this therapeutics, and a significant part of subjects may not respond or may develop resistance to their treatment. These unfavorable biological settings and unwanted consequences of treatments often are the causes that lead to disease progression and finally to death. The still unacceptable ratio of therapeutic failures urges us to develop novel approaches made steady on a more in‐depth knowledge of cancer biology and biochemistry, as well as improved therapeutics needed to address these challenges. In this context, cancer tissue stroma, which includes immune and endothelial cells, CAFs, and the ECM, plays an important role in tumor initiation, progression, metastatic spreading, and drug resistance.^35^, ^36^ To dismantle the shell wrapping the cancerous tissues made by the cancer‐associated fibrosis, several therapeutics acting on ECM components and/or fibroblasts have been proposed. For example, the combined use of FOLFIRINOX with a pegylated recombinant human hyaluronidase has been studied in a phase IB/II randomized study in patients with metastatic pancreatic adenocarcinoma.^37^ Unfortunately, the study outcome could not demonstrate any therapeutic improvements in comparison with FOLFIRINOX monotherapeutic regimen, but only showed the increase in unwanted side effects. Another tested approach was set up considering the use of FAK inhibitors,^38^ but the activation of a compensatory survival pathway that arises in pancreatic cancer cells made tumors resistant to therapy.^39^ The idea to deplete tumor stroma from CAFs to overcome cancer fibrosis was also investigated, by targeting FAP‐expressing cells, but FAP is not exclusively found in CAFs, and its expression by multipotent bone marrow stem cells and skeletal muscle implies that therapies targeting this protein can induce unwanted and potentially deleterious effects.^40^ In this context, the strategy of targeting BAG3 protein secreted by cancer cells seems to be promising for its effect in negatively modulating both TAMs and CAFs; it is worth noting that the amount of tumor stroma and collagen fibers is decreased in murine pancreatic cancer when treated with an anti‐BAG3 monoclonal antibody. The data here reported consolidate the hypothesis of the involvement of BAG3 protein in CAF activation and further clarify the correlation between *bag3* expression in patients affected by fibrotic tumors and their clinical outcome. Indeed, the analysis of PDXs databases allowed us to circumscribe and easier identify HNSCC, mesothelioma, and liver hepatocellular cancer as the fibrotic tumors presumably more responsive to the anti‐BAG3 therapy, due to the higher *bag3* expression levels that negatively correlate with patient survival. These findings foster the extension of the study about the role of this pathway in cancers of different origins and pave the way to a wider investigation on the use of the anti‐BAG3 strategy in fibrotic malignancies and diseases.

## CONFLICT OF INTERESTS

Margot De Marco, Anna Basile, Antonia Falco, Maria Caterina Turco, Alessandra Rosati, and Liberato Marzullo are shareholders of BIOUNIVERSA s.r.l.

## AUTHOR CONTRIBUTIONS

Margot De Marco, Nicoletta Del Papa, Maria Caterina Turco, Alessandra Rosati, and Liberato Marzullo conceived the project idea. Margot De Marco, Francesca Reppucci, Vittoria Iorio, Anna Basile, Antonia Falco, Roberta Iaccarino, and Sergio Brongo performed the experiments and analyzed the data. Margot De Marco and Nicoletta Del Papa drafted the manuscript and designed the figures. Maria Caterina Turco, Alessandra Rosati, and Liberato Marzullo revised the manuscript with input from Francesco De Caro and Mario Capunzo. All authors read and approved the final version of the manuscript.

## Data Availability

The data that supports the findings of this study are available from the corresponding author upon reasonable request.
